# Buried RF Sensors for Smart Road Infrastructure: Empirical Communication Range Testing, Propagation by Line of Sight, Diffraction and Reflection Model and Technology Comparison for 868 MHz–2.4 GHz

**DOI:** 10.3390/s23031669

**Published:** 2023-02-02

**Authors:** Vlad Marsic, Soroush Faramehr, Joe Fleming, Peter Ball, Shumao Ou, Petar Igic

**Affiliations:** 1 Centre for Advanced Low Carbon Propulsion Systems, Institute for Clean Growth and Future Mobility, Coventry University, Coventry CV1 5FB, UK; 2 School of Engineering, Computing and Mathematics, Oxford Brookes University, Wheatley Campus, Wheatley, Oxford OX33 1HX, UK; 3 School of Engineering, Computing and Mathematics, Faculty of Technology, Design and Environment, Oxford Brookes University, Wheatley Campus, Wheatley, Oxford OX33 1HX, UK

**Keywords:** RF, 868 MHz, 2.4 GHz, road sensors, road studs, car detection, smart infrastructure, buried sensors

## Abstract

Updating the road infrastructure requires the potential mass adoption of the road studs currently used in car detection, speed monitoring, and path marking. Road studs commonly include RF transceivers connecting the buried sensors to an offsite base station for centralized data management. Since traffic monitoring experiments through buried sensors are resource expensive and difficult, the literature detailing it is insufficient and inaccessible due to various strategic reasons. Moreover, as the main RF frequencies adopted for stud communication are either 868/915 MHz or 2.4 GHz, the radio coverage differs, and it is not readily predictable due to the low-power communication in the near proximity of the ground. This work delivers a reference study on low-power RF communication ranging for the two above frequencies up to 60 m. The experimental setup employs successive measurements and repositioning of a base station at three different heights of 0.5, 1 and 1.5 m, and is accompanied by an extensive theoretical analysis of propagation, including line of sight, diffraction, and wall reflection. Enhancing the tutorial value of this work, a correlation analysis using Pearson’s coefficient and root mean square error is performed between the field test and simulation results.

## 1. Introduction

**Figure 1:**
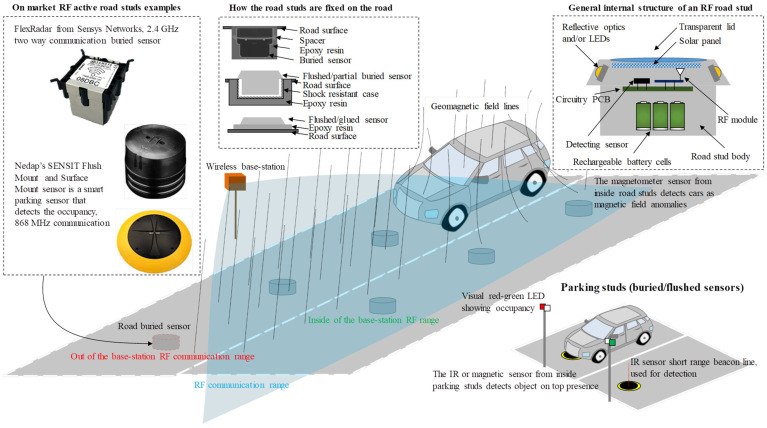
Road studs’ main applications for traffic monitoring and parking occupancy: market models examples, road mounting techniques, and general internal structure.

The smart vehicle revolution has started by pointing out the necessity of reducing exhaust gas emissions and improving drivers’ ability to react safely in dense traffic while finding an optimal route to their destination. Current infrastructure enables intelligent traffic lights and signaling using buried inductive loops [1] and highly elevated proximity sensors and video cameras [2] only mentioning the main used procedures. However, as a result of thermal stress and road vibration, the induction loops can fail due to wires breaking, whereas the proximity and video sensors are extremely sensitive to harsh weather conditions such as rain, snow, fog, etc. Consequently, novel approaches, such as buried and flush-mounted sensors called road studs, have been developed [3,4,5,6] to overcome the current equipment’s mentioned hazards. Besides the different approach towards the rough environmental conditions, the road studs can also provide a more elegant, less disruptive, and more aesthetic solution for speed monitoring and traffic density recording when compared with the temporary method of using piezo sensors activated by pneumatic tubes [2] or, can enhance the overall detection portfolio by the possible addition of pedestrians [7]. Regarding the vehicles’ static time, although the in-out time counting is provided by cameras with number recognition installed at the entrance and exits of the parking system of crowded cities or multistory buildings, road flush-mounted sensors are used to detect occupancy [8,9] while advertising the results on a high visibility position via a color based system. Last but not least, road lane marking areas with no street lighting during the night can be enhanced with smart flushed sensors equipped with solar panels and LEDs, delivering a reliable sensing and marking [10,11]. Figure 1 illustrates the main applications of the smart road studs; presents specific examples of RF studs technologies such as those provided by Sensys Networks [12,13] and NEDAP [14,15]; describes the most commonly used methods for mounting the studs on the ground’s surface; outlines a general RF smart stud internal structure.

As the costs and structure of road studs may be the main considerations and drivers for their mass adoption when compared with traditional road marking and signaling methods, recent advancements in technologies composing the road sensors may also play a catalyst part in their evolution and implementation. Current progress in the automotive electric sector and its supporting technologies may have indirect benefits for the large-scale introduction of future road studs:

- Embedded magnetic sensors could make road stud models more effective, sensitive, cheap, and robust if they are repurposed from their high-power applications [16] that are focused on inverters and solar power plants industry [17]. In addition, varying the magnetic sensor’s substrate materials, such as Si, SiC, GaN, may benefit from new simulation and analysis methods, which are currently specific only to those technologies [18,19].
- Multi-standard compliant RF transceiver chips may be employed for data links between sensors and an off-road base station. This way, by using modern multi-frequency wireless modules designed for smart electronics and inter-vehicle communication [20,21,22], future addition of road nodes and alternative data protocols bridging different networks together may be available.
- Despite the fact that road studs without rechargeable capabilities use rechargeable battery packs due to their high energy density, such as Li-ion technology, this energy storage technology can be improved furthermore by using battery cell internal instrumentation [23,24] specifically designed for automotive and off-grid energy storage applications.
- The newly developed IoT and 5G network [25] may be adjusted to cover data links for the remote road studs while, in parallel, providing real-time information to the moving vehicles.

Regardless of the current and upcoming technological advancements, since the buried sensors are installed at ground level, their useful RF coverage range between their locations and elevated base stations continues to be a challenging issue for a wide variety of fields [26,27,28,29,30,31,32]. Moreover, as most of the data available on road studies comes from companies’ marketing campaigns instead of research studies since traffic monitoring is a very challenging, hazardous, and expensive operation requiring efforts sometimes beyond academic institutions’ reach, the published information is scarce. Besides the low amount of low power propagation close to the ground experimental data, the accompanying theoretical support is insufficient as it requires diffraction addition to the other encountered phenomena. Therefore, it may be necessary to perform further modeling and analysis to investigate near-ground propagation beyond the simple path loss model [33] derived from Friis’ formula [34] and visual data interpretation.

This work’s aim is to provide an RF communication ranging reference for the 868 MHz and 2.4 GHz radio low-power propagation close to the ground by delivering a detailed set of signal measurements up to 60 m accompanied by suitable modeling and performing a thorough analysis. To enable the comparison and also to comply with the RF local standards for low-power free-license communication, both transmitting powers have been set at 0 dBm, same modulation scheme and message payload. Furthermore, a theoretical model including line of sight (LOS), reflection, and diffraction is derived while its suitability and possible applications are discussed in depth. An extended comparison and analysis of experimental results for the 868 MHz and 2.4 GHz frequencies between each other and compared with simulation results, respectively, is presented as a rigorous example before summarizing the main findings of this study.

Although buried sensors may be typically designed to monitor vehicles and space occupancy, they may yield real potential and strategic benefits in other fields such as defense, agriculture, geology, and seismology, as well as for hazardous locations. They may play a very important role in the future smart road infrastructure, detecting and guiding vehicles while marking lanes and indicating pedestrians on busy roads.

The work is structured into five main sections. The Section 1 mentions and explains the main categories of RF road studs considered in this study. It also depicts the possible drivers that may accelerate the large-scale adoption of the buried road sensors and states the objective of this study: to reference and evidence practically and theoretically the RF communication range for two free license frequencies of 868 MHz and 2.4 GHz, currently adopted by the RF road stud sector. In Section 2, where the study’s setup and methodology are presented, is further divided into three subsections: the Section 1 describes the experimental method and utilized equipment; the Section 2 provides the theoretical background, and this study’s considered variables. The Section 2.3 provides succinct schematics of the overall parameters accounted for the empirical and theoretical tests. Section 3 presents the results obtained during subsequent experiments from theoretical modeling and empirical testing. Section 4 analyses and comments on the previously presented results. The analysis starts by interpreting the resulted RF communication ranges and then, by employing basic statistical tools exemplifying how a rigorous interpretation of the resulted data may be contextually delivered. Section 5 presents the work’s main conclusions and mentions the possible fields and areas that may benefit from this RF low power close to ground propagation reference study.

## 2. Materials and Methods

### 2.1. Practical Testing Setup

The RF communication ranging tests have been carried out using a TI evaluation RF kit SMARTRF TRXEBK [35]. The RF kit includes two main sensor boards and four interchangeable separate daughter boards containing two pairs of the wireless transceiver CC1200 [36] for 868 MHz and the other two equipped with a 2.4 GHz CC2520 [37] transceiver, as used in [38,39]. The communication ranging tests are performed the same way for both tested frequencies. The transmitter (Tx) is placed on a tripod whose height is varied at 0.5, 1 and 1.5 m above the ground, emulating the off-road base station or the roadside unit’s (RSU) possible configurations, for which it is recommended to find the optimal elevation according to the application [40]. Alternatively, the buried receiving (Rx) sensor is housed in a transparent plastic box along with its electrical circuitry and antenna. To reduce the possible circuitry board’s scattering and reflections during the experiment, the whole Rx system has been accommodated inside an enclosed tin box equipped with a narrow opening on one side for the antenna and the USB cables. As a means of ensuring stable communication between Tx and Rx, both modules are powered via USB instead of batteries, which also provide the serial link for data transmitting settings, packet receiving, and recording. It was determined that 0 dBm is the common transmission power for both wireless communications at 868 MHz and 2.4 GHz [41,42,43] and that the GFSK modulation scheme could be used on both frequencies under IEEE 802.15.4 standard. Setting the payload to 30 Bytes emulates a possible road stud data frame which contains, for example, a detection time stamp (e.g., 3 Bytes), a vehicle type (e.g., 1 Byte), and detection-lost timestamps (e.g., vehicle leaving the sensor). This adopted test frame length is equivalent to up to four times the necessary energy for one transmission without draining the battery of the low-powered sensor, which is designed to operate for 2 to 10 years [44]. This assumption considers only the road stud’s active time after waking up from sleep mode by detecting a car via its onboard sensors. The reception and transmission of a data frame require an important amount of total stud’s power, approx. 55 mW [36,37], while the onboard sensor detection and microcontroller computations require at list one order lower than the Tx-Rx communication power [6,45,46].

**Figure 2:**
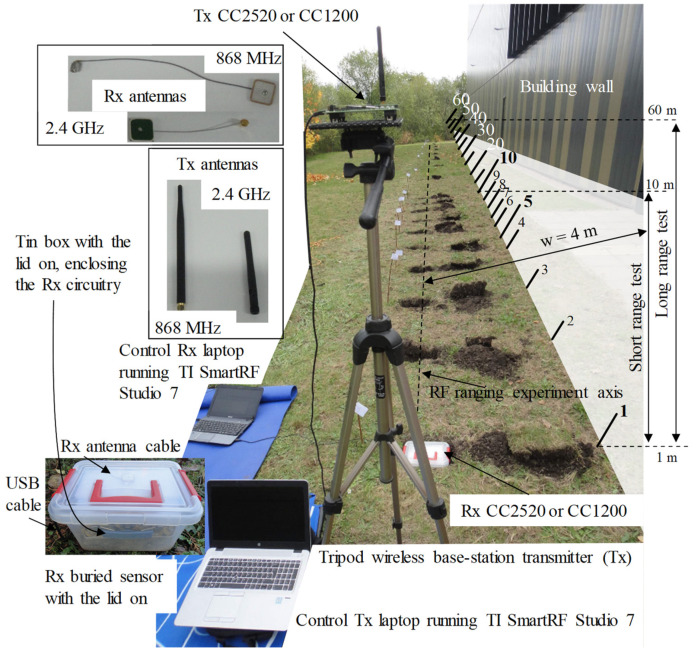
Experimental setup for the RF communication ranging between the Tx on tripod emulated base station and Rx buried sensor box, on 868 MHz and 2.4 GHz. The holes are distanced from Tx fixed location from 1 to 60 m, the correct elevation of the Rx being achieved with foam layers inside the main plastic box.

In the evaluation of data, three scenarios have been considered: short-range 1 to 10 m with a step of 1 m; long-range 1 to 60 m with a step of 5 m; and mixed, which is a combination of the previous two scenarios. The setup background includes an adjacent wall parallel to the ranging communication distance, whose width (w) from the RF sensor testing axis is 4 m, which is a common distance found in densely populated cities where the first lane of the street is at 3–4 m from the surrounding fences, walls, and other obstacles. Figure 2 illustrates the testing setup and the sensor configurations for Tx and Rx, along with the antennas used during the experiments. For the Tx cases, two dipole wipe antennas were used separately for 868 MHz [47] and 2.4 GHz [48], while for the buried sensor Rx, two ceramic patch antennas matched for 868 MHz [49] and 2.4 GHz [50] were employed. Since in real life, the RSU and the RF road studs are abiding by the 802.15.4 standard; the communication is bidirectional; an acknowledgment for each received frame is sent back to the Tx. Moreover, many times, the RSU sends a puling data request to the stud to initiate communication. Therefore, any scenario, such as when the stud only receives or only transmits data to RSU, can be considered a representative testing evaluation of a real-life scenario. Another testing consideration regarding the road stud real-time or real-life functioning scenario is that it does not transmit data when a vehicle is on top, or it does while the RSU is installed in a favorable spatial position for receiving. Measures were taken to prevent optimal communication in the testing scenario, therefore when later improvements may be required, changing these initial parameters potentially data link performance to be achieved:

- For 2.4 GHz IEE 802.15.4, the eleventh transmission channel was selected; this does not exclude WiFi interference, which can be obtained by selecting channels 15, 16, 21, and 22 for Europe or 15, 20, 25, and 26 for North America [51].
- Both data rates are set to 150 kbps in the 868 MHz band and 250 kbps in the 2.4 GHz band, the top level outlined in IEEE 802.15.4 [52]; it is well known that a high data rate reduces RF ranges.
- The RF buried test is set on wet soil instead of tarmac, the water concentration in the material around the RF sensor being an important attenuation factor as specified in [53]. Consequently, placing it on concrete or tarmac later could reduce attenuation and improve communication.
- A generic right-handed circular polarized (RHCP) antenna is used on the Rx sensor, such as those used in GPS systems where the Tx uses a left-handed circular polarized (LHCP) antenna, while the Tx used in this setup uses a linear polarized antenna. Due to the fact that the Tx may only cover a roadside network without a mesh network surrounding it, a directional high-gain antenna may be more effective than the omnidirectional ones used in this study in terms of data link and gain.
- As the Rx antennas are equipped with U.FL connectors, and the TI transceiver is equipped with SMA type, a U.FL to SMA adapter has been used. This introduces insertion losses, which can be eliminated by a modified design with soldered antenna connections.
- The testing Tx transmission power is set at 0 dBm for both communication frequencies, although higher levels are permitted, and they can improve the RF data link if required.

The Tx is set to continually transmit while the Rx is set to receive only 100 packets at each testing hole location. Employing this method, a maximum (Max), minimum (Min), as well as an average (Avg) received signal strength indicator (RSSI) can be calculated and used in the analysis in conjunction with the received messages containing cyclic redundancy checks (CRC) errors and lost packages due to other errors (Lost). For the experiments, the box containing the Rx sensor is buried, and its top lid is exposed at ground level. Between the ceramic antenna patch and the circuitry enclosing the tin box of the Rx, foam layers are placed to adjust the antenna level below the plastic lid.

The Rx box’s plastic lid is on for all RF testing duration and acts similar to a radar antenna’s radome, protecting the antenna and circuitry from outside harsh weather, although inducing some signal attenuations. The attenuations are to be considered when the radome thickness *th* (i.e., in the specialized literature, the radome thickens is abbreviated by the *t* symbol; however, to not be confused with time notation, this study will use *th* instead) is on the same scale with the communication wavelength *λ.* However, when *th* is much smaller than *λ*, the induced losses are sub-unitary [54,55,56]. In this study, the plastic lid thickness is 1 mm, while *th* represents the 2 cm distance of the Rx antenna to the lid; therefore, for the testing *λ* of 0.345 and 0.124 m, the lid-induced losses can be neglected since the weather during experiments was dry (i.e., without inducing water accumulations on the lid area).

### 2.2. RF Scenario’s Propagation Overall Theoretical Considerents

**Figure 3:**
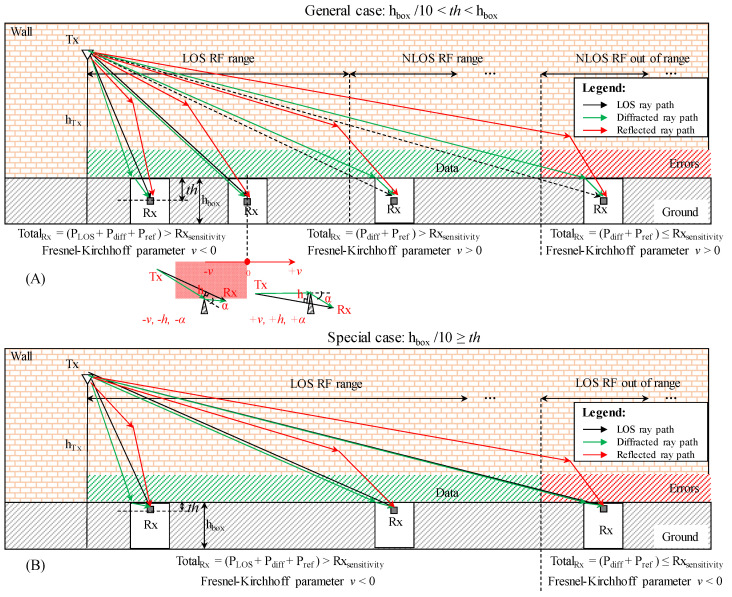
Illustration of the general case for RF communication ranging between an elevated transmitter (Tx) and a buried receiver (Rx) sensor showing the breaking point when the direct line of sight (LOS) *d*_1_ overlaps with the diffracted path *d*_3_ when Rx’s antenna distance to the enclosure’s top lid *th* is large when compared with box’s height *h_box_* (**A**). The special case is when *th* is one order smaller than *h_box_*, and due to this relationship, the breaking does not occur (**B**). Both cases take account of diffraction and reflection, while the diffraction parameter *v* is used to highlight the difference between the two scenarios.

It is good practice for a test setup to start with the correct assertion, such as the theoretical scenario, the experiment’s initial conditions, boundaries, and effects accounted for in the previous section. Figure 3 depicts the general propagation case for a buried sensor equipped with an internal antenna (Figure 3A) and the special case when the antenna is positioned beneath the box’s top lid (Figure 3B). General and special cases differ mainly in how they combine the line of sight (LOS) and non-line of sight (NLOS). The two cases of LOS and NLOS start simultaneously and then split sequentially for the general case (Figure 3A), whereas they remain together until the end of the special case (Figure 3B). The general scenario’s splitting place is determined by the origin point of Fresnel-Kirchhoff diffraction parameter *v*, which characterize the diffraction propagation. The diffraction parameter’s sign is according to the resultant height *h* and diffraction angle *α* signs: when *h* is above the knife-edge and *α* ϵ [ 0°, 180°), then *v*, *h*, and *α* are negative, and when *h* is below the knife-edge, and *α* ϵ [ 180°, 0°), *v*, *h*, and *α* are positive [57]. Due to the parallel wall that runs along the RF communication ranging setup, the reflected rays add up to the total received power, which needs to be higher than the Rx sensitivity in order to distinguish the signal from the background noise without errors. In this setup, the Rx sensitivity for 868 MHz is −107 dBm [36] and −98 dBm for 2.4 GHz [37]. Since the Rx antenna in this work is placed immediately below the buried box’s top lid, the LOS ray path will contribute to the whole RF communication range while *v* will vary in the negative interval, as shown in Figure 3B.

By excluding the summation generalization from the overall formula presented in [58], we can compute the total received signal on the Rx side according to our presented scenario:

(1) $$r{\left(t\right)}_{total}=Re\{\frac{\lambda }{4\pi }[\frac{g_{LOS}\sqrt{G_{0}}}{d_{1}}u\left(t-\tau_{0}\right)e^{-j2\pi d_{1}\frac{1}{\lambda }}+\frac{g_{diff}\sqrt{G_{d}}}{d_{21}+d_{22}}u\left(t-\tau_{d}\right)e^{-j2\pi [d_{1}-\left(d_{21}+d_{22}\right)]\frac{1}{\lambda }}+\frac{g_{ref}\sqrt{G_{r}}}{d_{31}+d_{32}}u\left(t-\tau_{r}\right)e^{-j2\pi [d_{1}-\left(d_{31}+d_{32}\right)]\frac{1}{\lambda }}]e^{j2\pi ft}\}$$

where *G*_0_, *G_d_*, and *G_r_* are the product of Tx and Rx antenna gains on direction of the LOS, diffracted and reflected path, *τ*_0_ = *d*_1_/*c*, *τ_d_* = (*d*_21_ + *d*_22_)/*c* and *τ_r_* = (*d*_31_ + *d*_32_)/*c* are the LOS, diffraction and reflection induced delays, *u*(*t*) is the complex signal function such as *Ae-*^*j*(*ωt*+*φ*)^ with *A* signal’s amplitude, *ω* = 2*πf* angular frequency, *t* standing for time and *φ* for phase. The complex signal, if required, may support the Gaussian frequency-shift keying (GFSK) modulation as implemented in [38]. The *λ* is the wavelength associated accordingly to each of the two frequencies *f* used in this study. The gains for LOS, diffraction, and reflection are represented by *g_LOS_*, *g_diff_*, and *g_ref_*, respectively.

In light of the fact that this scenario schematic displays a broad view of the key phenomena that need to be considered, understanding the modeling requires an individual geometrical approach to each gain, which is discussed in the following sections.

#### 2.2.1. RF Line of Sight Propagation

**Figure 4:**
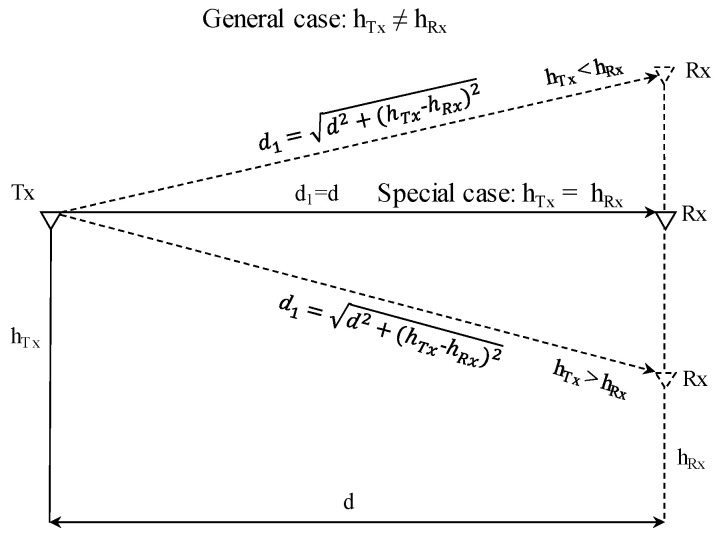
The line of sight (LOS) *d*_1_ path calculation considerations for the general case when Tx height differs from Rx elevation and for the special case occurs when both are at the same level.

As there are no obstacles, there is only free space propagation until it reaches Rx, the LOS component *d*_1_ in our ray model has a significant contribution to the received signal’s power. It is straightforward to calculate *d*_1_, as shown in Figure 4, and it should be noted that *d*_1_ *= d* only when Tx and Rx are at the same height. Therefore, the received signal can be calculated as follows:

(2) $$r{\left(t\right)}_{LOS}=Re\left\{\frac{\lambda }{4\pi }\left(\frac{g_{LOS}\sqrt{G_{0}}}{d_{1}}\right)u\left(t-\tau_{0}\right)e^{j2\pi ft}\right\}$$

In this case, the well-known the Friis transmission formula can be used to define *g_LOS_*:

(3) $$g_{LOS}=\frac{\lambda \sqrt{G_{0}}}{4\pi d_{1}}$$

Multiplying this expression with the first term in Equation (1) and we will obtain the general expression for LOS free space path loss (*FSPL*) formulation, which shows the transmitted power normed by the received power, *P_t_*/*P_r_*:

(4) $$FSPL=\frac{P_{t}}{P_{r}}={\left(g_{LOS}\frac{\lambda \sqrt{G_{0}}}{4\pi d_{1}}\right)}^{-1}=\frac{1}{G_{0}}{\left(\frac{4\pi d_{1}}{\lambda }\right)}^{2}$$

Various times *G*_0_ is considered 1 dBi such as for ideal dipole or monopole antennas and the *FSPL* expression is transformed from W to dB as *FSPL* = 10log_10_(4*πd*_1_/*λ*)^2^ and applying the logarithmic operations for powers results the well-known form of 20log_10_(4*πd*_1_/*λ*) [59]. It is important to note that *FSPL* is always positive. On the other hand, path gain (*PG*) is typically described as a negative term. In [58], *PG* is defined as *PG* = *P_r_*/*P_t_* linearly, while logarithmic *PG* is defined as *PG* = −*FSPL*. In practice, however, it is generally referred as path loss and used positively or negatively accordingly.

#### 2.2.2. RF Diffraction Propagation

**Figure 5:**
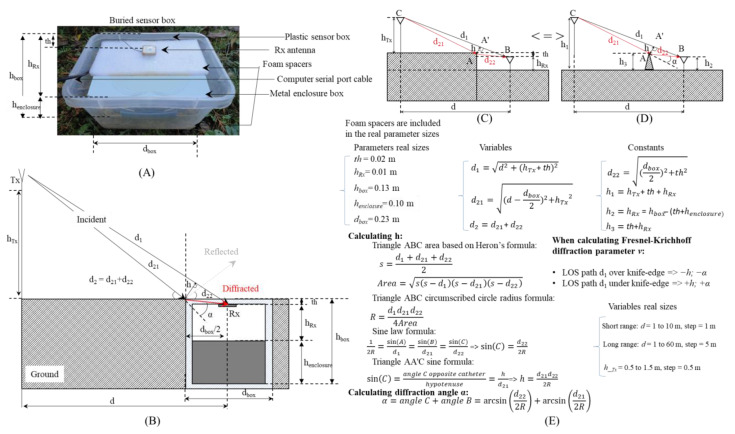
Buried sensor’s box and its associated dimensions (**A**), the theoretical considerations (**B**), the scenario transformation from terrain diffraction (**C**) to terrain knife-edge diffraction model (**D**), and the explicit modeling parameters (**E**).

A schematic representation of the experimental setup and the RF propagation associated with this study’s scenario is shown in Figure 5. The sensor box dimensions are represented on the test box (Figure 5A), while the overall modeling scenario involving the two ray paths *d*_1_ for LOS and *d*_2_ for diffracted NLOS is shown in (Figure 5B). A detailed description of the parameters that govern the equivalent model transformation from the ground-diffracted signal (Figure 5C) to knife-edge diffraction (Figure 5D) is found in the geometrical relationships noted in (Figure 5E). Among the parameters presented in (Figure 5E), there are three columns: first, the associated dimensions; second, the variables that change with different Tx-Rx distances; and third, the constants not affected by distance variance.

To calculate the propagation associated with the knife-edge diffraction, the formulation given in [58,60] is adopted:

(5) $$\Delta d=\frac{h^{2}}{2}\frac{d_{21}+d_{22}}{d_{21}d_{22}}$$

with the phase difference between the LOS path *d*_1_ and diffracted path *d*_2_:

(6) $$\Delta \varphi =\frac{2\pi \Delta d}{\lambda }=\frac{\pi }{2}v^{2}$$

where *λ* is the signal’s wavelength and *v* is the Fresnel–Kirchhoff diffraction parameter:

(7) $$v=h\sqrt{\frac{2\left(d_{21}+d_{22}\right)}{\lambda d_{21}d_{22}}}$$

The diffraction parameter *v* is inheriting the sign of *h* and diffraction angle *α* as explained previously and illustrated in Figure 3.

The normalized electric field at the Rx relative to the LOS *d*_1_ path becomes [60]:

(8) $$\frac{E_{diff}}{E_{LOS}}=F\left(v\right)=\frac{1+j}{2}\int_{v}^{\infty }e^{-j\frac{\pi }{2}s^{2}}ds=\frac{1+j}{2}\left[\int_{v}^{\infty }\cos\left(\frac{\pi s^{2}}{2}\right)ds-j\int_{v}^{\infty }\sin\left(\frac{\pi s^{2}}{2}\right)ds\right]=C\left(v\right)-jS\left(v\right)$$

where *F*(*v*) is the complex Fresnel integral defined as the union of its two parts *C*(*v*) and *S*(*v*), derived via Euler’s formula, where *s* is a dummy variable replacing *v* in order that *F* be expressed through it. One of the most important properties of *C* and *S* is:

(9) $$\{\begin{array}{c} \int_{v}^{\infty }\cos\left(\frac{\pi s^{2}}{2}\right)ds=\int_{0}^{\infty }\cos\left(\frac{\pi s^{2}}{2}\right)ds-\int_{0}^{v}\cos\left(\frac{\pi s^{2}}{2}\right)ds\\ C\left(\infty \right)=S\left(\infty \right)=\frac{1}{2} \end{array}$$

and based on (9) the normalized electric field can be expressed as:

(10) $$\frac{E_{diff}}{E_{LOS}}=F\left(v\right)=\frac{1+j}{2}\left[\left(\frac{1}{2}-C\left(v\right)\right)-j\left(\frac{1}{2}-S\left(v\right)\right)\right]$$

Lee’s approximations [61] for the diffraction gain in decibels *g_diff_* = 20log_10_|*F*(*v*)| [62] simplifies Fresnel’s complex integral computation for a known *v*:

(11) $$g_{diff}\approx \{\begin{array}{cc} 0\;\mathrm{dB}, & v<-1\\ 20\log_{10}\left(0.5-0.62v\right)\;\mathrm{dB}, & -0.8\leq v<0\\ 20\log_{10}\left(0.5e^{-0.95v}\right)\;\mathrm{dB}, & 0\leq v<1\\ 20\log_{10}\left(0.4-\sqrt{0.1184-{\left(0.38-0.1v\right)}^{2}}\right)\;\mathrm{dB}, & 1\leq v\leq 2.4\\ 20\log_{10}\left(\frac{0.225}{v}\right)\;\mathrm{dB}, & v>2.4 \end{array}$$

It could be feasible to use Lee’s approximation to estimate Fresnel’s integral as a scalar, since it only needs to be evaluated on one diffraction point at Rx. The received diffraction-induced signal at the Rx side can be expressed as follows:

(12) $$r{\left(t\right)}_{diff}=Re\left\{\frac{\lambda }{4\pi }\left[\frac{g_{diff\_linear}\sqrt{G_{diff}}}{d_{21}+d_{22}}u\left(t-\tau \right)e^{-j2\pi \left(d_{1}-\left(d_{21}+d_{22}\right)\right)\frac{1}{\lambda }}\right]e^{j2\pi ft}\right\}$$

It should be mentioned that if Lee’s approximations are to be used in Equation (12), the *g_diff_* will need translation from logarithm to linear:

(13) $$g_{diff\_linear}=10^{\frac{g_{diff}}{10}}$$

#### 2.2.3. RF Communication Reflection Propagation

In this study, the reflection case can be considered as a two-ray model scenario since a Tx–Rx direct path and a Tx–Wall–Rx reflected path can be accounted together beside the previous discussed diffraction case; therefore, by following the general formula of given by [58], the path loss for LOS has already been included:

(14) $$r{\left(t\right)}_{TwoRay}=r{\left(t\right)}_{LOS}+r{\left(t\right)}_{ref}=Re\left\{\frac{\lambda }{4\pi }\left[\frac{g_{LOS}\sqrt{G_{0}}}{d_{1}}u\left(t-\tau_{0}\right)e^{-j2\pi d_{1}\frac{1}{\lambda }}+\frac{g_{ref}\sqrt{G_{r}}}{d_{31}+d_{32}}u\left(t-\tau_{r}\right)e^{-j2\pi \left(d_{1}-\left(d_{31}+d_{32))}\right)\right)\frac{1}{\lambda }}\right]e^{j2\pi ft}\right\}$$

where the received signal at Rx accounting only for reflection is:

(15) $$r{\left(t\right)}_{ref}=Re\left\{\frac{\lambda }{4\pi }\frac{g_{ref}\sqrt{G_{r}}}{d_{31}+d_{32}}u\left(t-\tau_{r}\right)e^{-j2\pi \left(d_{1}-\left(d_{31}+d_{32}\right)\right)\frac{1}{\lambda }}e^{j2\pi ft}\right\}$$

The reflection gain *g_ref_* is Fresnel’s reflection coefficient which generally is split between its complex polarizations vertical (*R_V_*) and horizontal (*R_H_*) expressions. The modulus reflection coefficient (*R*) which, for the purpose of this work, will be considered a general measure of the reflection’s magnitude:

(16) $$\{\begin{array}{c} g_{ref}=R=\left|R_{V}+R_{H}\right|=\sqrt{R_{V}^{2}+R_{H}^{2}}\\ R_{V}=\frac{\eta \sin\left(\beta \right)-\sqrt{\eta -\cos^{2}\left(\beta \right)}}{\eta \sin\left(\beta \right)+\sqrt{\eta -\cos^{2}\left(\beta \right)}}\\ R_{H}=\frac{\sin\left(\beta \right)-\sqrt{\eta -\cos^{2}\left(\beta \right)}}{\sin\left(\beta \right)+\sqrt{\eta -\cos^{2}\left(\beta \right)}}\\ \eta =\varepsilon_{r}^{*}=\frac{\varepsilon^{*}}{\varepsilon_{0}}=\varepsilon_{r}-j\frac{\sigma }{\omega \varepsilon_{0}}=\varepsilon_{r}-j\frac{Z_{0}}{2\pi }\lambda \sigma \approx \varepsilon_{r}-j60\lambda \sigma \end{array}$$

where *η*, *ε_r_*, and *σ* are the material’s complex relative permittivity (i.e., also known as the dielectric constant), relative electric permittivity and conductivity while ε* is the material complex permittivity [63,64]; *β* is the reflection or the grazing angle from the wall and *ω* = 2*πc*/*λ* is the angular frequency. In this study, *ε_r_* and *σ* values were chosen according to [53] for metal, as the wall is covered with decorative metal panels. It is noted that sometimes the material refractive index *n* is found instead of *η*, the relationship between them being given by free space impedance: *Z*_0_ = *nZ*, *Z* representing the material impedance and replaced in Equation (16).

Finally, the overall power received at the Rx side *P_r_*, after considering the transformations from [58], is as follows:

(17) $$P_{r}=P_{0}P_{t}{\left[\frac{\lambda }{4\pi }\right]}^{2}{\left|\frac{\sqrt{G_{0}}e^{-j\Delta \varphi_{1}}}{d_{1}}+\frac{g_{dif\_linear}\sqrt{G_{d}}e^{-j\Delta \varphi_{2}}}{d_{2}}+\frac{R\sqrt{G_{r}}e^{-j\Delta \varphi_{3}}}{d_{3}}\right|}^{2}$$

with the simplifications:

(18) $$\{\begin{array}{c} \Delta \varphi_{1}=\frac{2\pi d_{1}}{\lambda }\\ \Delta \varphi_{2}=\frac{2\pi (d_{1}-d_{2})}{\lambda }\\ \Delta \varphi_{3}=\frac{2\pi (d_{1}-d_{3})}{\lambda }\\ d_{2}=d_{21}+d_{22}\\ d_{3}=d_{31}+d_{32} \end{array}$$

*P_0_* represents an adjustment of data to real communication conditions based on the maximum power level indicated by the receiver close to the transmitter, since, except for high-end radio equipment, no receiver will indicate 0 dBm when at the same height, immediate after the transition zone in far field. This is due to the high-end RF equipment using received signal strength (RSS) instead of the RSSI which is an 8-bit quantization of the received frame preamble bits’ power for 802.15.4 standard [65]. For high-frequency tests, as presented in this study, starting close to 1 GHz, *P*_0_ is usually between −10 and −40 dBm for 868 MHz and 2.4 GHz, as the first highest value from the 255 values specified for RSSI in the datasheet. Although there will be a shift down of the whole simulation data series on the y-axis (i.e., RSSI scale), it will emulate the pattern better than the hypothesis of 0 dBm initial receiving levels.

**Figure 6:**
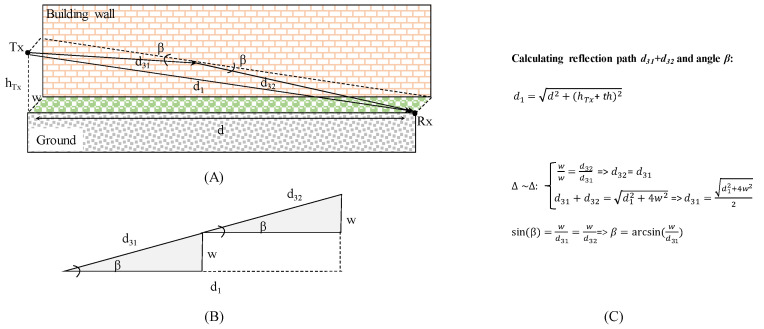
Wall reflection path (**A**), similar triangles generally used for reflection path distance calculation (**B**), and geometrical relationships delivering reflection angle *β* and reflection path *d_31_ + d_32_* (**C**).

The geometrical considerations and calculations for the reflected path *d*_3_ are shown in Figure 6.

As another consideration when comparing simulation data with measurement values, if the empirical data is expressed in dBm as is the case for RSSI, then the final transformation for the received power should be logarithmic in nature and on the dBm scale as follows:

(19) $$P_{r\_dBm}=10\log_{10}\left(P_{r}\right)+30$$

### 2.3. Considered Parameters for Experimental and Theoretical Tests and Analysis

**Figure 7:**
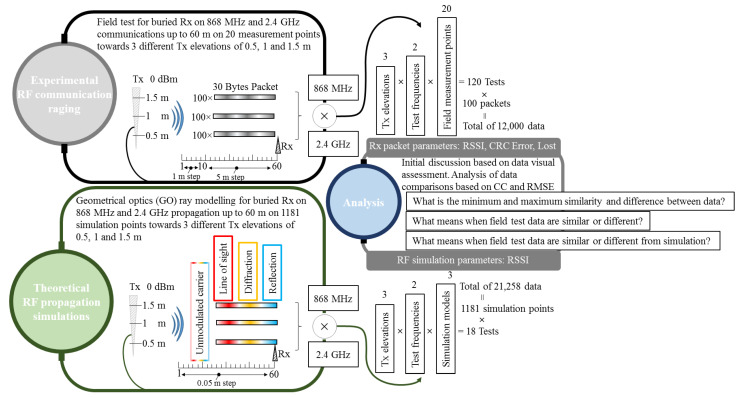
Overall considered parameters for empirical, simulation, and data analysis used in this study. The total results produced by experimental and simulation setups provide the analysis parameters. Although the correlation coefficient (CC) and root mean square error (RMSE) are applied only on the received signal strength indicator (RSSI), the other two package’s parameters, such as cyclic redundancy check (CRC) error and lost messages, are used to assert the overall signal quality at certain measurement points.

As this study exploits various scenarios and requires a large collection of parameters to analyze the RF low power propagation close to the ground level, an overall visual schematic is presented in Figure 7 to deliver a clear picture regarding the links between experimental results and analysis.

It is worth noting that the wet soil in which the sensors were buried will be implicitly included only in the field test experiment’s results and not in the theoretical models. On the theoretical side, since for the ray model, only one reflection instead of multiple ones are considered, and the Rx is placed at the surface level, the soil reflection does not contribute, while only the reflection from the metal side wall is accounted. In addition, since the diffraction is on one point (i.e., perpendicular to the measurement hole’s wedge), it becomes independent from the material’s properties, as the opposite when it warps as Keller’s cone for wedge diffraction [66,67]. Moreover, since refraction was not included in the GO ray model due to the soil’s potential inhomogeneity and unknown structure, the soil’s water content contribution is not accounted for by any of the phenomena participating in the overall simulation results.

To summarize, a theoretical demonstration for this study’s wet soil attenuation is not appropriate. However, RF measurement studies suggest that dry construction materials attenuate the microwave spectrum less than when water-saturated [68,69], which supports our hypothesis regarding wet soil attenuation disadvantaging the RF propagation.

## 3. Results

The propagation model described in the previous section is implemented via MATLAB, displaying the total received power at Rx as a cumulated result of LOS, diffraction, and reflection. In the end part of the Section 3, the simulation outcome illustrating all accounted phenomena through their total contribution will be replotted as dotted lines for comparison with the average RSSI values of the empirical measurements. The average RSSI values of the empirical measurements will be represented as bar graphs to highlight the difference from previous graphs, where only simulated or only measurement results were plotted at once.

The RSSI test measurement data are displayed for both frequencies at three Tx different elevations on their medium resultant value. Each graph includes on top a table indicating the maximum, minimum, and average RSSI encountered for 100 received packets at each Rx testing hole. The table inclusion was adopted instead of the graph’s variance bars since, for the 2.4 GHz frequency, the erroneous and lost packets are accounted as well to complete the overall communication picture.

### 3.1. LOS Path-Loss, Knife Edge Diffraction, and Reflection Simulation Results

**Figure 8:**
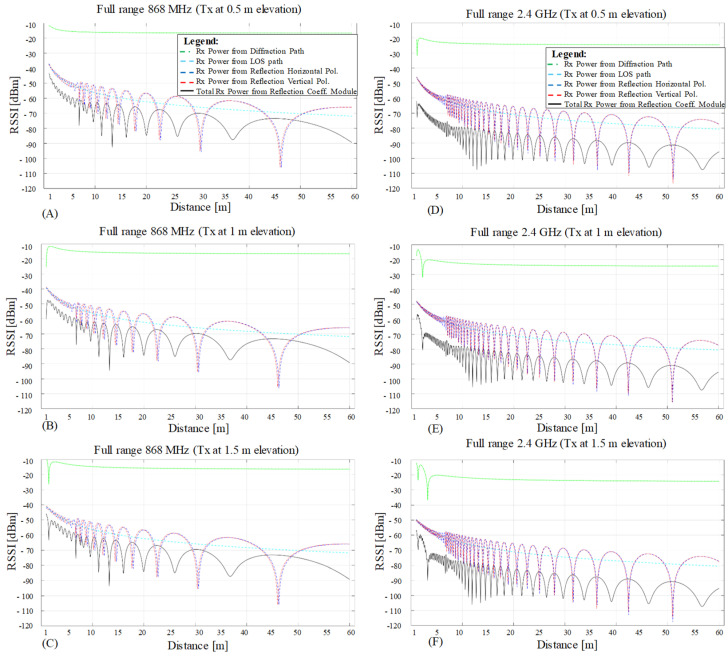
Simulation results illustrating each individual component of the received power for the mix range from 1 to 60 m at three different Tx elevations: 0.5 m (**A**,**D**), 1 m (**B**,**E**), and 1.5 m (**C**,**F**) on the test frequencies of 868 MHz (**A**–**C**) and 2.4 GHz (**D**,**E**). The total power is plotted with continuous black line to illustrate the end result of all participating factors. Since the Tx or Rx distance to the side wall does not change when varying the Tx elevation, the reflection and direct path contributions on each frequency are similar. The variation can be observed in the diffraction plot (green dotted line), as Tx elevation varies the incident angle and the distance path of the simulated phenomena.

Figure 8 illustrates the independent simulation values for LOS (cyan line), diffraction (green line), horizontally polarized reflection (blue line), and vertically polarized reflection (red line), plotted for three different Tx elevations on frequencies of 868 MHz (Figure 8A–C) and 2.4 GHz (Figure 8D–F). The considered modeling interval is the union mix range of the previous two ranges from 1 to 60 m. Each phenomenon contributes a specific amount to the total received power (black line), as demonstrated in this plot. The resolution step used for the simulated results is 5 cm resulting in a total of 1181 data points from 1 to 60 m domain. This allows us to follow all the received signal power variations that cannot be extrapolated when displayed on only 20 points, such as in the case of overlapping the empirical test points for comparison purposes.

The propagation with reflection includes the LOS contribution as well, and it is the same for all three Tx elevations on each frequency since the Tx or Rx distance to the reflective side wall does not change. The received power delivered via reflection and LOS can change significantly when elevation is varied, such as for reflective ground or ceiling if actively acting in the RF experiment; however, for this presented study, only the side wall contributes through reflection. Nevertheless, each graph shows a different diffraction contribution due to Rx’s position change reflected on the diffraction’s interference fringe minima on the short-range up to 5 m, since the incident angle varies, decreasing proportionally with the increasing distance towards Tx.

### 3.2. Empirical Testing

**Figure 9:**
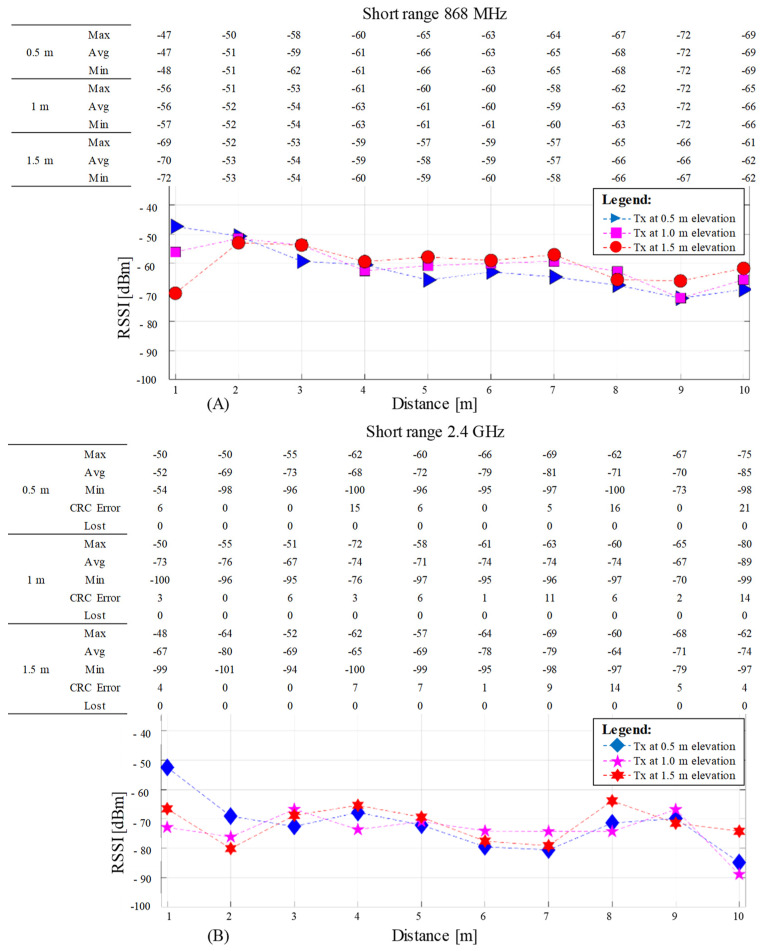
RF communication ranging RSSI results for short range from 1 to 10 M with 1 m step between measurement points, at 868 MHz (**A**) and 2.4 GHz (**B**). Each graph has on top a table indicating the minimum (Min), maximum (Max), and average (Avg) RSSI values. The 2.4 GHz table contains two additional rows indicating the number of received packets containing CRC errors (CRC Error) and the considered lost packages due to other errors (Lost). The 868 MHz does not have the last two rows since no errors were recorded.

Figure 9 shows the results of short-range measurements from 1 to 10 m, with one-meter steps between testing points at 868 MHz (Figure 9A) and 2.4GHz (Figure 9B). Each graph shows three different series, each corresponding to different Tx elevations of 1.5, 1, and 0.5 m above ground level. The graphs are accompanied by a table showing the maximum, average, and minimum RSSI values for each measurement point from 100 received packages. Additionally, in the table for 2.4 GHz is the number of received packets with CRC errors and the number of lost packets. The 868 MHz did not experience any receiving errors; therefore, these fields are not shown in its associated table.

**Figure 10:**
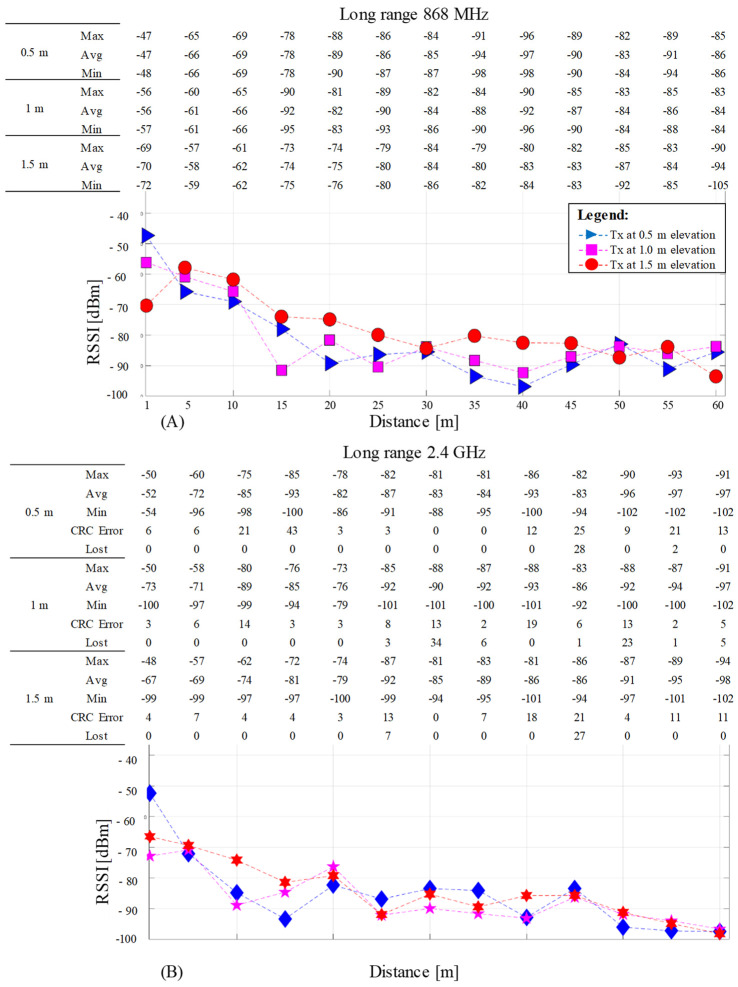
RF communication ranging RSSI results for long range from 1 to 60 m with 5 m step between measurement points, at 868 MHz (**A**) and 2.4 GHz (**B**). Each graph has on top a table indicating the minimum (Min), maximum (Max), and average (Avg) RSSI values. The 2.4 GHz table contains two additional rows indicating the number of received packets containing CRC errors (CRC Error) and the considered lost packages due to other errors (Lost). The 868 MHz does not have the last two rows since no errors were recorded.

The long-range results, Figure 10, are shown for a distance from 1 to 60 m with a 5 m step between the measurement holes at 868 MHz (Figure 10A) and 2.4 GHz (Figure 10B). The graphs and the accompanying tables use the same arrangement as previously presented in Figure 9 for consistency.

### 3.3. Average RSSI Measurements Versus Simulation Results

**Figure 11:**
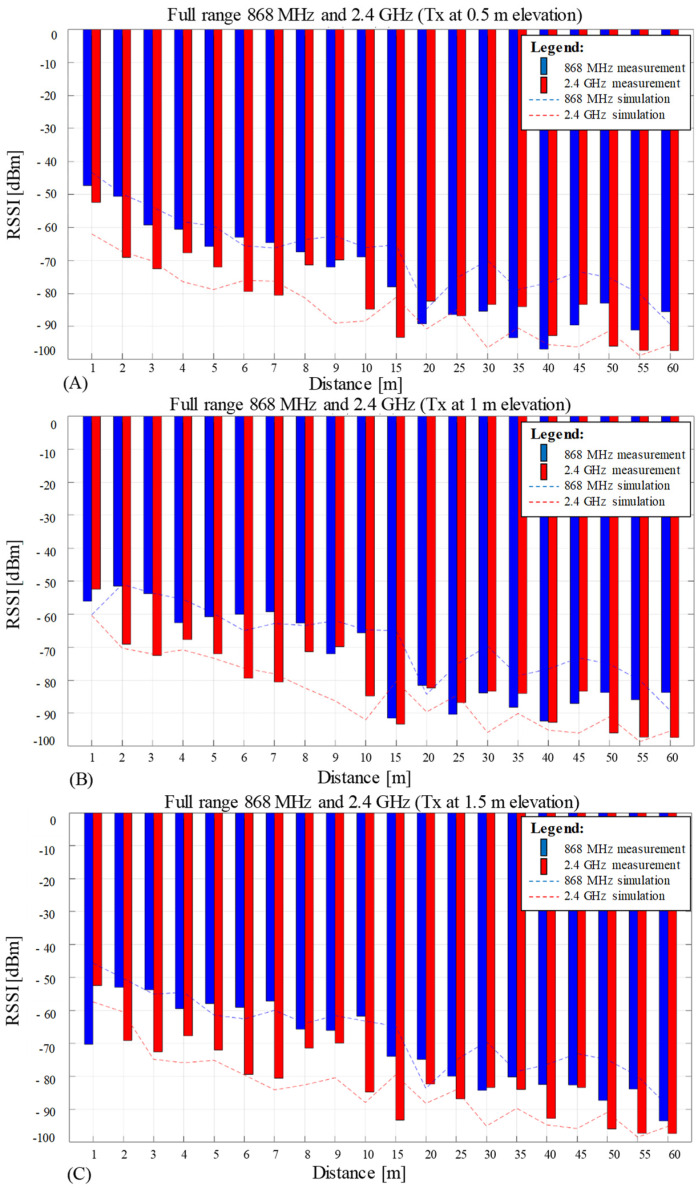
Measurement average RSSI values (bar graph) versus simulation results for path loss with diffraction (dotted lines) for three different Tx elevations: 0.5 m (**A**), 1 m (**B**), and 1.5 m (**C**).

Figure 11 illustrates the average RSSI values plotted for both frequencies of 868 MHz and 2.4 GHz on the mix range of measurements from 1 to 60 m, each graph illustrating the results for one single Tx elevation: 0.5 m (Figure 11A), 1 m (Figure 11B) and 1.5 m (Figure 11C). The experimental results are represented as bars, blue for 868 MHz and red for 2.4 GHz.

Two dotted lines representing the simulation’s total received power results, including LOS, diffraction, and reflection, are overlaid on the bar plot’s experimental values. Each line presents a different color associated with the simulated frequencies: blue for 868 MHz and red for 2.4 GHz. The simulation of the total received power for both frequencies contains only 20 points; this low resolution matches the experimental data for comparison reasons. Therefore, the plots of the simulation dotted lines show now, as expected, abrupt and truncated transitions between consecutive values when compared with the smooth graph resulting from 1181 data points, displayed previously in Figure 8.

## 4. Discussion

For the same Tx power while using similar transmitting and receiving antennas, it was expected that 868 MHz would show a greater range than the 2.4 GHz counterpart. From the initial setup, the data rate for 868 MHz was 100 kbps lower than the 2.4 GHz, a factor that also translates into a higher for the 868 MHz coverage since the data density is lower. However, since the measurements were performed at close proximity to the ground for the Tx’s elevation while Rx was 2 cm below the surface level (i.e., *th* = 0.02 m), the performance at 868 MHz, due to its larger wavelength compared with 2.4 GHz, was clearly disadvantaged at 0.5 m Tx elevation. This is due to the Tx at 868 MHz being still in the transition zone rather than far field due to its 0.345 m wavelength, whereas, for 2.4 GHz (i.e., 0.125 m wavelength) the 0.5 m elevation represents 4*λ* which is equivalent to the far-field region.

Although the results are showing coverage for short and long ranges on both frequencies while the simulation model visually agrees well with the empirical results, a more comprehensive comparison can be performed by employing the root mean square error (RMSE) and Pearson’s correlation coefficient (CC). An RMSE value of zero indicates identical data, whereas any other value on a dBm scale indicates disagreement between the two compared sets. The CC’s values range from minus one to one, with zero representing no agreement, one indicating a strong positive relationship, and minus one indicating a strong negative relationship. In this work, the average RSSI for 868 MHz and 2.4 GHz compared with the simulation’s data is presented in Table 1. Although the overall RMSE between the simulation model and the average RSSI measurements is between 7 and 10 dBm, the overall CC is above 75%. This indicates a satisfactory agreement between measurements and simulations, with the exception of 2.4 GHz at 1.5 m, where the correlation is moderate at 66%. Since there are only 20 measuring points for each data set, if only five of them are not matching, then a 25% disagreement is immediately observed despite empirical data following the model. Furthermore, the compared RSSI data are averaged values based on 100 reception packages, and the 66% moderate correlation result also reflects the CRC errors and lost frames for 2.4 GHz.

For comparing only the reception quality between 868 MHz and 2.4 GHz, the overall RMSE and CC are presented in Table 2. As can be observed, the RMSE disagreement between 868 MHz and 2.4 GHz is approximately 11 dB for each Tx elevation. Across all sets of tests, this relates to an overall correlation of more than 75%. Similar to the previous comparison of measurements and simulations, this result suggests there is no more than a 25% difference between reception for the two technologies. It follows that 868 MHz transmissions near the ground deteriorate more than 2.4 GHz since 868 MHz is expected to offer a notable improvement in RF coverage when compared with 2.4 GHz.

Table 3 shows individual RMSE and CC for the full mixed range of 1 to 60 m. Worth to be noted that the table’s RMSE analysis is focusing on each measurement point of the two testing frequencies disregarding the overall measurement and simulation trends for the data sets. It can be observed that the maximum disagreement between the two communication frequencies propagating close to the ground occurs at 1.5 m Tx elevation and on the 2 m Rx’s test hole. When investigated further with the aid of simulation results from Figure 11, it reveals that this 2 m measurement discrepancy happens on all three Tx elevation scenarios. Therefore, the maximum RMSE between the two different frequencies at 2 m hole indicates a normal propagation case, where the tested wavelengths and distances are delivering the RSSI in accordance with propagation theory for RF in the far field. Alternatively, the minimum disagreement between the two frequencies occurs at the 25 m test point and 0.5 m Tx elevation. The results demonstrate that the performance at 868 MHz is reduced at low angles and low heights above ground and becomes more similar to 2.4 GHz behavior, as shown by the perfect overlap between the RSSI data for 2.4 GHz and simulation data for the same frequency, Figure 11A. Although that 2.4 GHz at 25 m recorded 20% erroneous packets, Figure 10B, this may be corrected next time by modifying accordingly the parameters that were set initially to not favor a good reception as enumerated in Section 2.1 Practical testing setup.

## 5. Conclusions

This work illustrates a comprehensive experimental and theoretical radio coverage study addressing potential RF sensors located below the ground surface capable of communicating with an elevated Tx base station. Three different positions of the Tx elevation have been used to emphasize the influence of the ground’s proximity on wireless communications. A simulation ray model including reflection, diffraction, and LOS has been developed and compared with the empirical results. Next, the simulation and experimental results have been analyzed using the statistical tools delivered by RMSE and CC, identifying the similarities and differences between 868 MHz and 2.4 GHz. The overall results show that a transmission range of up to 60 m is possible at both 868 MHz and 2.4 GHz, while the simulation and experimental sets display a good correlation outlining this detailed study’s outcome.

The work provides a reference by delivering real measurement data, modeling, and analysis for communication domains that are currently investigating the potential exploitation of radio frequency buried sensors. Moreover, a comparative study between the most commonly used free license communication frequencies, 868 MHz and 2.4 GHz, is presented, providing the RF research community with valuable data in this area where the information is not abundant due to various strategic reasons. The rigorous analysis and interpretation of the results may serve as a model for future comparisons between simulation and measurement values since many studies confine themselves to just visual interpretation of data. In light of the potential applications of using buried sensors, the results of this study will be useful not only for intelligent transportation systems but also for agriculture, geology, mining, and other areas that monitor and investigate the ground surface remotely.

## Figures and Tables

**Table 1:** Overall (RMSE) and Correlation Coefficient (CC) between average RSSI test values and total received power model, including LOS, diffraction, and reflection, for three different Tx elevations.

Tx Elevation [m]	Frequency [MHz]	Overall RMSE [dBm]	Overall CC [%]
0.5 m	868	9.56	0.89
	2400	10.06	0.79
1 m	868	8.43	0.8
	2400	8.26	0.77
1.5 m	868	7.49	0.75
	2400	9.36	0.66
Tx to Rx distance [m]		1 to 60	1 to 60

**Table 2:** Overall (RMSE) and Correlation Coefficient (CC) between the average RSSI test values at 868 MHz and 2.4 GHz for three different Tx elevations.

Tx Elevation [m]	Overall RMSE [dBm]	Overall CC [%]
0.5 m	10.48	0.79
1.0 m	11.84	0.78
1.5 m	11.46	0.78
Tx to Rx distance [m]	1 to 60	1 to 60

**Table 3:** Individual (RMSE) between the average RSSI test values at 868 MHz and 2.4 GHz for three different Tx elevations.

Tx elevation [m]	Individual RMSE calculated on the average RSSI values resulted from the measurements at 868 MHz and 2.4 GHz [dBm]																			
0.5 m	5.08	18.4	13.2	7.2	6.32	16.5	15.8	3.84	2.18	15.8	15.3	6.89	0.46	2.06	9.47	4.07	6.32	13	6.06	11.8
1.0 m	16.7	24.6	12.9	11	10	14	14.9	11.5	5.24	23.1	6.98	5.34	1.64	5.98	3.33	0.7	0.79	8.09	7.95	12.8
1.5 m	3.81	27.1	15	5.9	11.5	18.5	22	1.83	5.35	12.4	7.42	4.35	12	0.91	9.17	3.17	3	3.86	10.9	4.6
Tx to Rx distance [m]	1	2	3	4	5	6	7	8	9	10	15	20	25	30	35	40	45	50	55	60

## Data Availability

The datasets generated and analyzed during this study are available from the corresponding author on a reasonable request, but restrictions apply to the commercially-confident details.
